# Timeliness of vaccine administration among children in urban informal settlements in Nairobi, Kenya

**DOI:** 10.1371/journal.pgph.0005462

**Published:** 2026-02-11

**Authors:** Maurine Ng’oda, Jonathan Izudi, Collins Omenda, Anne Njeri, Nelson Mbaya, Abdhalah Ziraba

**Affiliations:** 1 Emerging and Re-emerging Infectious Diseases Unit, African Population and Health Research Center, Nairobi, Kenya; 2 Department of Community Health, Faculty of Medicine, Mbarara University of Science and Technology, Mbarara, Uganda; 3 Infectious Diseases Institute, Makerere University College of Health Sciences, Kampala, Uganda; 4 Directorate of Graduate Training, Research and Innovation, Muni University, Arua, Uganda; Murdoch Children's Research Institute, AUSTRALIA

## Abstract

Timeliness of vaccination among children in urban informal settlements is understudied in sub-Saharan Africa. We determined the proportion of children below 5 years who received vaccines on time and the associated factors in two large urban informal settlements in Nairobi, Kenya. We conducted an analytic cross-sectional study in Viwandani and Korogocho, randomly selected households with a child below 5 years, and administered questionnaires to mothers/caregivers. Timely vaccination was defined according to the Kenya Expanded Programme on Immunization schedule. Vaccination was considered timely if administered within 28 days of birth for Bacille Calmette-Guérin (BCG), between 14 and 18 weeks for Diphtheria-Tetanus-Pertussis-Haemophilus influenzae type b-Hepatitis B dose 3 (DTP-Hib-HepB-3), and between 9 and 10 months for Measles-Containing Vaccine dose 1 (MCV1). Multivariable modified Poisson regression identified the factors associated with timely vaccination. Of 412 children, 216 (52%) with verifiable immunization booklets were analyzed for timeliness. Timely vaccination rates were 90.4% for BCG (189/209), 90.2% for DTP-HIB-Hep-3 (184/204), and 84.2% for MCV1 (160/190). Factors associated with timely vaccination included caregiver uncertainty about access to health services, which reduced the likelihood of timely BCG (adjusted prevalence risk ratio [aPR] 0.87, 95% confidence interval [CI] 0.78-0.96), DTP-Hib-HepB-3 (aPR 0.88, 95% CI 0.79-0.98), and MCV1 (aPR 0.81, 95% CI 0.70-0.94). Compared with Korogocho, children in Viwandani were more likely to receive timely MCV1 (aPR 1.18, 95% CI 1.03-1.35), whereas children of Christian caregivers were less likely than those of non-religious caregivers (aPR 0.83, 95% CI 0.70-0.99). Overall, vaccination timeliness varied by antigen, with a slight decline over time for later-schedule vaccines such as MCV1. Residence, religion, and access to routine health services were key determinants of timely vaccination. Strengthening outreach, faith-based engagement, and reminder systems in informal settlements like Korogocho could enhance vaccine timeliness, particularly for vaccines administered later in infancy.

## Introduction

Timely vaccination is the cornerstone of immunization programs, ensuring optimal protection against vaccine-preventable diseases during the early years of a child’s life [1]. Children under five are highly vulnerable to severe infections, complications, and death from vaccine-preventable diseases such as tuberculosis, diphtheria, pertussis, tetanus, and measles [2]. Timely immunization is important to catalyze herd immunity, curb disease transmission, and protect those who remain unvaccinated [3]. Delays in vaccination increase the risk of morbidity and mortality and exacerbate vaccine-preventable disease outbreaks, as earlier reported in multiple measles and pertussis outbreaks resulting from missed vaccination schedules in sub-Saharan Africa [2]. Vaccination delays prolong periods of susceptibility to vaccine-preventable diseases and may compound under-immunization in marginalized populations.

Urban informal settlements face unique challenges that disrupt vaccine timeliness, including financial barriers and constrained healthcare services [4]. Systemic inequities expose children in these settings to vaccine-preventable diseases, as structural barriers—such as distant health facilities, under-resourced clinics, and healthcare worker shortages—further hinder timely immunization [4]. Additionally, caregivers in these informal settings often lack awareness of immunization schedules or face competing socio-economic priorities, such as daily wage labor, that delay healthcare-seeking [5]. These challenges are exacerbated by the underrepresentation of the growing urban informal settlement populations in national surveys, including studies on routine immunization. Demographic and Health Surveys, including the recent (2022) Kenya Demographic and Health Survey (KDHS), have historically excluded informal settlements or relied on broad “urban poor” indicators to assess immunization program performance. This limits the accuracy of immunization data in such settings [6]. Indicators from other urban areas often obscure the complex inequities faced by informal settlement residents and overlook critical factors affecting healthcare access and behavior. Without precise, disaggregated data from urban informal settlements, routine vaccination interventions risk being ineffective or misaligned with the community’s needs.

In Kenya, localized data such as that provided by the Nairobi Urban Health and Demographic Surveillance System (NUHDSS) have been instrumental in highlighting immunization trends in marginalized populations. For instance, while overall immunization coverage has improved over the years due to targeted health interventions [7], previous studies have revealed persistently delayed vaccinations, driven by demand-side barriers such as caregiver education, and supply-side challenges, such as vaccine stockouts [8]. This underscores the complexity of improving vaccine timeliness in vulnerable populations.

The timely administration of Bacille Calmette-Guérin (BCG), Diphtheria-Tetanus-Pertussis-Haemophilus influenzae type b-Hepatitis B dose 3 (DTP-Hib-HepB-3), and Measles-Containing Vaccine dose 1 (MCV1) vaccines in informal settlements is critical, as these vaccines collectively indicate key dimensions of routine immunization performance and healthcare access. Timely BCG vaccination at birth reflects the coverage of skilled birth attendance, given that the vaccine is typically administered immediately after delivery, and the effectiveness of linkage between communities and formal health facilities. BCG vaccination within four weeks of birth further reflects timely access to routine immunization services. Timely DTP-Hib-HepB-3 vaccination demonstrates the health system’s capacity to retain infants in the immunization schedule and the acceptability of vaccination services among caregivers. Lastly, timely MCV1 vaccination at nine months signifies the health system’s ability to sustain care continuity and the overall performance of immunization programs. Examining the timeliness of these vaccines in informal settlements, where health inequities and access barriers are prevalent, provides insights into delays in childhood immunization. The study, therefore, aimed to assess the timeliness of BCG, DTP-HIB-Hep-3, and MCV1 administration, as well as the determinants, among children in two urban informal settlements in Nairobi, Kenya.

## Methods and materials

### Setting and study design

We analyzed data from the Kenya Multisite Integrated Serosurveillance (KEMIS) project. KEMIS was conducted from September 2020 to June 2024 within the Nairobi Urban Health and Demographic Surveillance System (NUHDSS), and included four cross-sectional surveys carried out between March and May 2021, March and June 2022, September and December 2022, and July and October 2023. In this study, we analyzed data from Korogocho and Viwandani informal settlements.

### Study population and sampling

At each survey round, we randomly selected an individual per household from the NUHDSS population register, totaling 850 individuals. We stratified the sample by age group to achieve representativeness of the broader NUHDSS population structure. For individuals aged 15–64 years, and those aged 65 and above, 50 participants were selected from each 5-year age band, while for those aged 0–14 years, 100 participants were selected from each 5-year age band. This means that by design, only one eligible child was selected per household. This approach minimized clustering effects and ensured a more diverse household-level sample.

### Data collection

All caregivers of randomly selected children under five years (N = 412) were interviewed, regardless of vaccination card availability. Accurate timing could not be established using maternal recall alone. We also considered age eligibility based on Kenya’s Expanded Program on Immunization (KEPI) schedule [9]. A total of 196 children, representing 48% of the original sample, were excluded because they lacked vaccination cards. From the 216 who had immunization booklets, records with missing vaccination dates for any of the three target vaccines were excluded only from the analysis of the specific vaccine concerned. For instance, a child with a recorded date for MCV1 but not for BCG was retained in the overall study sample but excluded from the BCG-specific timeliness analysis. After applying these inclusion and exclusion criteria, only eligible children per each antigen were included in the final analysis.

### Variables and measurements

#### Outcome variable.

For each antigen, the vaccination status was verified using routine immunization cards. We defined timeliness based on KEPI’s recommended vaccination ages [9], allowing a 28-day grace period beyond the recommended schedule, consistent with previous studies on vaccination timeliness [10,11]. In Kenya, the recommended vaccination schedule includes BCG at birth, DTP-Hib-Hep-3 at 14 weeks, and MCV1 at 9 months. Accordingly, vaccination was considered timely if administered within 28 days of birth for BCG, between 14 and 18 weeks for DTP-HIB-Hep-3, and between 9 and 10 months for MCV1. Vaccine administration timelines were measured on a binary scale, with timely vaccination coded as 1 and untimely vaccination as 0.

#### Independent variables.

The independent variables in this study included maternal, child, and health system factors, along with contextual factors such as study site and survey round. The maternal variables included religion and educational level. Religion was categorized as none, Muslim, or Christian, while maternal education was coded as a binary variable distinguishing between no formal education and primary or higher education. The child-related variables included sex and age. Age was measured as a continuous variable and summarized using the mean and standard deviation, whereas sex was coded as male or female. The health system’s related variables included missing antenatal care attendance during the previous pregnancy, missing institutional delivery, and inability to access routine healthcare services during the COVID-19 pandemic in the last 6 months. Antenatal care attendance was measured by asking whether the mother had missed any antenatal visits during her last pregnancy, coded as yes, no, or do not know.

Missing institutional delivery was also coded as yes, no, or do not know. The inability to access routine healthcare services during the COVID-19 pandemic in the last six months was measured on a binary scale as yes or no. Contextual factors included the study site and survey round. The study site was categorized on a binary scale as either Korogocho or Viwandani, while the survey round variable was measured based on the timing of data collection across four rounds, labelled as Round 1 through Round 4.

### Statistical analysis

We summarized sociodemographic characteristics using descriptive statistics and calculated the proportion of children who received the vaccines on time, expressed as a percentage. We assessed associations between covariates and timely vaccination using Chi-square and Fisher’s exact tests for categorical variables. We also conducted a sensitivity analysis to assess potential selection bias by comparing baseline characteristics between included and excluded records. We identified the factors associated with timely vaccination using a modified Poisson regression model with robust standard errors since the outcome was common. Prevalence risk ratios (PRRs) with corresponding 95% confidence intervals were reported for each association. The final model included a priori selected factors known from the literature [10,11] to influence vaccination timeliness—including maternal, child, health system, and contextual factors, as well as those found statistically significant in the bivariable analysis. All analyses were conducted in Stata version 15 (StataCorp, College Station, TX), and statistical significance was set at p < 0.05.

### Ethical considerations

We obtained ethical approval for the study from the Kenya Medical Research Institute Scientific and Ethics Review Unit (KEMRI/SERU/CGMR-C/203/4085) and secured written informed consent from mothers and caregivers aged 18 years and above. Informed consent was required to ensure that the mother or caregiver understood the study’s purpose, procedures, risks, and benefits, and voluntarily agreed to participate on behalf of the child.

### Inclusivity in global research

Supporting Information (S1 File) shows information regarding ethical, cultural, and scientific considerations specific to inclusivity in global research.

## Results

### Characteristics of study participants

**Fig 1:**
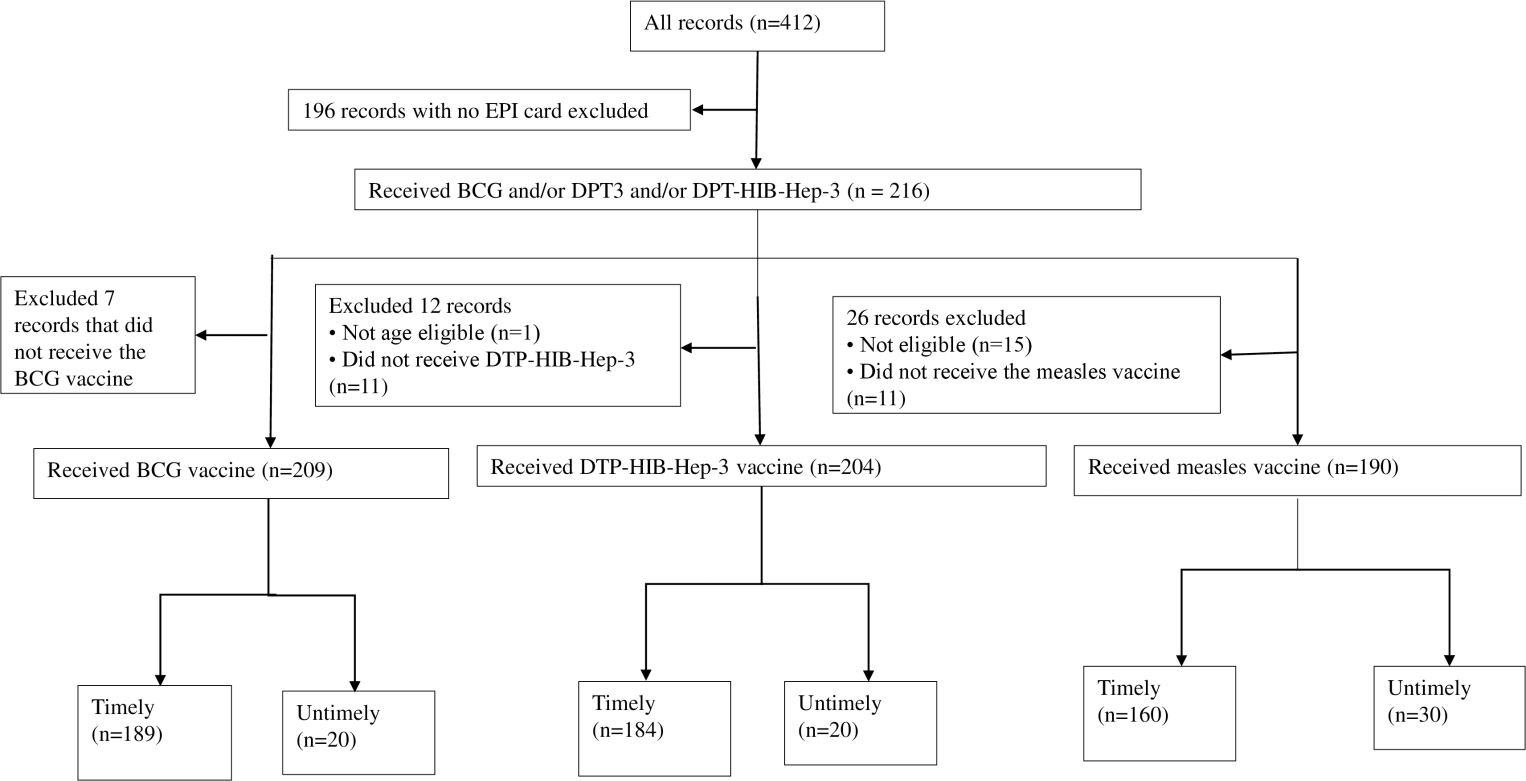
Flow diagram showing the characteristics of study participants. The majority of caregivers (85%) identified as Christians, and more than half of the mothers (57%) had no formal education. The sex distribution of the children was nearly balanced across all vaccine groups, with 51% male and 49% female.

After applying the inclusion and exclusion criteria (Fig 1), out of the 216 records, 209 children were included in the BCG analysis, following exclusion of seven who had no dates for BCG administration. The DTP-HIB-Hep-3 analysis included 204 children, following the exclusion of 12, of whom 11 had no date records and one was not age-eligible. For the MCV1 analysis, 190 children were included after excluding 26, of whom 15 were not age-eligible, and 11 had no record of dates in their booklets.

### Comparison of included and excluded records

We compared socio-demographic and health system–related characteristics between included (n = 216) and excluded (n = 196) records (S1 Table). Our results showed that the two groups were similar concerning the study site, child sex, religion, maternal education, and antenatal care attendance (all p > 0.05). However, we observed systematic differences between the excluded and included records regarding the survey round and access to routine health services during the COVID-19 pandemic (all p < 0.05). Caregivers more frequently reported uncertainty about access to routine health services. In addition to caregivers with excluded records reporting higher uncertainty regarding access to routine health services during COVID-19 compared to those with included records (52.3% vs 47.7%), they reported higher inability to access services (56.3% vs 43.8%).

### Bivariate analysis of differences in timely BCG, DTP-HIB-Hep-3, and MCV1 vaccination

Table 1 shows differences in the timeliness of BCG, DTP-HIB-Hep-3, and MCV1 vaccination in the bivariate analysis. Among the 209 children who received BCG, 189 (90.4%) received the vaccine on time.

**Table 1:** Bivariate analysis of differences in timely BCG, DTP-HIB-Hep-3, and MCV1 vaccination.

Variables	Level	BCG (n = 209)			DTP-HIB-Hep-3 (n = 204)			MCV1 (n = 190)		
	Timeliness	No 20 (9.6)	Yes 189 (90.4)	P-value	No 20 (9.8)	Yes 184 (90.2)	P-value	No 30 (15.8)	Yes 160 (84.2)	P-value
Religion	None	0 (0.0)	3 (100.0)	1.00	0 (0.0)	3 (100.0)	0.54	0 (0.0)	3 (100.0)	0.47
	Muslims	1 (6.3)	15 (93.8)		0 (0.0)	15 (100.0)		4 (26.7)	11 (73.3)	
	Christians	19 (10.0)	171 (90.0)		20 (10.8)	166 (89.2)		26 (15.1)	146 (84.9)	
Highest level of maternal education	No formal education	16 (8.5)	173 (91.5)	0.11	16 (8.7)	168 (91.3)	0.12	27 (15.7)	145 (84.3)	0.91
	Primary and higher	4 (20.0)	16 (80.0)		4 (20.0)	16 (80.0)		3 (16.7)	15 (83.3)	
Sex	Female	13 (12.5)	91 (87.5)	0.15	11 (10.8)	91 (89.2)	0.64	19 (20.4)	74 (79.6)	0.09
	Male	7 (6.7)	98 (93.3)		9 (8.8)	93 (91.2)		11 (11.3)	86 (88.7)	
Survey round	Round 1	8 (25.8)	23 (74.2)	0.02	7 (24.1)	22 (75.9)	0.06	11 (37.9)	18 (62.1)	0.01
	Round 2	4 (7.7)	48 (92.3)		3 (5.8)	49 (94.2)		5 (11.4)	39 (88.6)	
	Round 3	3 (4.8)	59 (95.2)		4 (6.7)	56 (93.3)		6 (10.3)	52 (89.7)	
	Round 4	5 (7.8)	59 (92.2)		6 (9.5)	57 (90.5)		8 (13.6)	51 (86.4)	
Study site	Korogocho	10 (12.3)	71 (87.7)	0.28	10 (12.8)	68 (87.2)	0.25	16 (22.6)	55 (77.4)	0.04
	Viwandani	10 (7.8)	118 (92.2)		10 (7.9)	116 (92.1)		14 (11.8)	105 (88.2)	
No access to routine health services during the COVID-19 pandemic in the last six months	Yes	2 (14.3)	12 (85.7)	0.21	1 (7.1)	13 (92.9)	0.16	2 (15.4)	11 (84.6)	0.14
	No	5 (5.1)	93 (94.9)		6 (40.0)	90 (60.0)		9 (10.3)	78 (89.7)	
	Do not know	13 (13.4)	84 (86.6)		13 (13.8)	81 (86.2)		19 (21.1)	71 (78.9)	
Missed antenatal care during the last pregnancy	Yes	0 (0.0)	1 (100.0)	1.00	0 (0.0)	1 (100.0)	1.00	0 (0.0)	1 (100.0)	1.00
	No	20 (9.6)	188 (90.4)		20 (9.8)	183 (90.2)		30 (15.9)	159 (84.1)	
Missed delivery at a health facility	No	20 (9.6)	189 (90.4)		7 (6.2)	105 (93.8)	0.06	11 (10.7)	91 (89.3)	0.04
	Yes	0 (0.0)	0 (0.0)		13 (62.0)	79 (38.0)		19 (21.6)	69 (78.4)	

Of these, 171 (90.0%) had mothers or caregivers who identified as Christian, 173 (91.5%) had mothers with no formal education, 98 (93.3%) were male, and 118 (92.2%) were from the Viwandani study site. For the 204 children who received DTP-HIB-Hep-3, 184 (90.2%) received it on time. Of these, 166 (89.2%) had mothers or caregivers who identified as Christian, 168 (91.3%) had mothers with no formal education, 93 (91.2%) were male, and 116 (92.1%) were from the Viwandani study site. Lastly, among the 190 children who received MCV1, 160 (84.2%) got it on time. Of these 146 (84.9%) had mothers or caregivers who identified as Christian, and 145 (84.3%) had mothers with no formal education. Additionally, most children who received the MCV1 on time were male and from the Viwandani.

Timeliness did not vary significantly by religion or maternal education level across the three vaccines. Most mothers (over 80%) had no formal education, and their children showed similar levels of timely vaccination compared to those whose mothers had at least primary education. Although slightly more male than female children were vaccinated on time, sex differences were not statistically significant across vaccine groups (p > 0.05). Timeliness differed significantly by survey round. For instance, the proportion of children vaccinated on time increased from 74.2% in Round 1 to 95.2% in Round 3 and the dropped to 92.2% in Round 4 for BCG (p = 0.02), and from 62.1% in Round 1 to 89.7% in Round 3 and then dropped to 86.4% in Round 4 for MCV1 (p = 0.01). Timeliness of MCV1 vaccination differed significantly by study site (p = 0.04). A higher proportion of children from Viwandani received MCV1 on time compared with those from Korogocho (88.2% vs. 77.4%). Regarding health system factors, timeliness was not significantly associated with missed antenatal care, delivery location, or access to routine health services during the COVID-19 pandemic.

### Factors associated with the timely BCG, DTP-HIB-Hep-3, and MCV1 vaccination in urban informal settlements in Nairobi, Kenya

Table 2 shows the factors associated with the timely administration of BCG, DTP-HIB-Hep-3, and MCV1. After adjusting for potential confounders (study site, sex, maternal education, religion, access to health services during the COVID-19 pandemic, and survey round), children living in Viwandani were significantly more likely to receive MCV1 on time compared to those in Korogocho (aPR 1.18, 95% CI 1.03-1.35). A similar, but statistically non-significant trend was observed for the BCG vaccine (aPR 1.09, 95% CI 0.99-1.2) and DTP-HIB-Hep-3 (aPR 1.10, 95% CI 0.99-1.21) vaccines. Male children compared with female children showed a trend toward a higher likelihood of timely vaccination, with aPR of 1.07 (95% CI 0.98–1.16) for BCG, aPR of 1.02 (95% CI 0.93–1.11) for DTP-HIB-Hep-3, and aPR of 1.11 (95% CI 0.98–1.25) for MCV1. Maternal education showed no significant association with timely vaccination. Compared with children whose mothers had no formal education, those whose mothers attained primary education or higher had slightly lower but not statistically significant likelihood of timely vaccination across all vaccines, with aPR of 0.83 (95% CI 0.67–1.05) for BCG, aPR of 0.85 (95% CI 0.68–1.06) for DTP-HIB-Hep-3, and aPR of 0.92 (95% CI 0.75–1.12) for MCV1.

**Table 2:** Factors associated with timely BCG, DTP-HIB-Hep-3, and MCV1 vaccination among children in urban informal settlements in Nairobi, Kenya.

		Timely administration of vaccines					
Variables	Level	BCG		DTP-Hib-Hep-3		MCV1	
		uPR (95% CI)	aPR (95% CI)	uPR (95% CI	aPR (95% CI)	uPR (95% CI	aPR (95% CI)
Study site	Korogocho	1	1	1	1	1	1
	Viwandani	1.05 (0.96 – 1.16)	1.09 (0.99 – 1.20)	1.06 (0.96 – 1.17)	1.10 (0.99 – 1.21)	1.14 (0.99 – 1.31)	1.18** (1.03 – 1.35)
Sex	Female	1	1	1	1	1	1
	Male	1.07 (0.98 – 1.17)	1.07 (0.98 – 1.16)	1.02 (0.93 – 1.12)	1.02 (0.93 – 1.11)	1.11 (0.98 – 1.26)	1.11 (0.98 – 1.25)
Highest level of maternal education	No formal education	1	1	1	1	1	1
	Primary and higher	0.87 (0.70 – 1.09)	0.83 (0.67 – 1.05)	0.88 (0.70 – 1.10)	0.85 (0.68 – 1.06)	0.99 (0.80 – 1.23)	0.92 (0.75 – 1.12)
Religion	None	1	1	1	1	1	1
	Muslims	0.94 (0.83 – 1.06)	0.87 (0.70 – 1.08)	1	0.90 (0.74 – 1.11)	0.73(0.54 – 1.00)	0.72 (0.51 – 1.02)
	Christians	0.90*** (0.86 – 0.94)	0.84 (0.69 – 1.02)	0.89*** (0.85 – 0.94)	0.81 (0.66 – 1.00)	0.85*** (0.80 – 0.90)	0.83** (0.70 – 0.99)
No access to health services during the COVID-19 pandemic in the last six months	No	1	1	1	1	1	1
	Yes	0.90 (0.73 – 1.12)	0.89 (0.71 – 1.11)	0.99 (0.85 – 1.16)	0.98 (0.83 – 1.14)	0.94 (0.74 – 1.20)	0.91 (0.72 – 1.15)
	Do not know	0.91(0.83 – 1.00)	0.87*** (0.78 – 0.96)	0.92 (0.83 – 1.01)	0.88** (0.79 – 0.98)	0.88 (0.77 – 1.00)	0.81*** (0.70 – 0.94)
Observations		209	209	204	204	190	190

Statistical significance code: *** p < 0.01, ** p < 0.05, * p < 0.1; All models adjusted for study site, sex, highest level of maternal education, religion, access to health services during the COVID-19 pandemic, and survey round; The adjustment for survey round was to control for time differences; Bolded figures are statistically significant at the 5% significance level; aPR: Adjusted prevalence risk ratio; uPR: Unadjusted prevalence risk ratio.

Religious affiliation, however, showed significant differences. Compared to children of caregivers with no religious affiliation, those from Christian households were significantly less likely to receive vaccines on time, with aPR of 0.84 (95% CI 0.69–1.02) for BCG, aPR of 0.81 (95% CI 0.66–1.00) for DTP-HIB-Hep-3, and aPR of 0.83 (95% CI 0.70–0.99) for MCV1. Children from Muslim households had a lower likelihood of timely MCV1 vaccination (aPR 0.72, 95% CI 0.51–1.02), though this association was not statistically significant. Children whose caregivers reported uncertainty (“do not know”) about access were significantly less likely to have timely vaccination across all vaccines, with aPR of 0.87 (95% CI 0.78–0.96) for BCG, aPR of 0.88 (95% CI 0.79–0.98) for DTP-HIB-Hep-3, and APR of 0.81 (95% CI 0.70–0.94) for MCV1.

## Discussion

We assessed the timeliness of administration of BCG, DTP-HIB-Hep-3, and MCV1 and the associated factors among children in two urban informal settlements (Korogocho and Viwandani) in Nairobi, Kenya. Timely vaccination was highest for BCG, followed by DTP-HIB-Hep-3, and lowest for MCV1. While previous studies suggest that caregivers may be more likely to adhere to vaccination schedules once routine immunization has started [12], our findings indicate that timeliness declines for later doses, likely due to factors such as increasing child age, family mobility within informal settlements, and missed opportunities at health facilities. The observed high timeliness for BCG may be attributed to its provision at birth or during early postnatal visits, as many deliveries appear to occur in health facilities. However, the observed decline in timeliness for later-schedule vaccines, particularly MCV1, reflects a pattern of declining attendance, which could be due to declining caregiver engagement with health services as the child grows older and the interval between vaccination visits increases, or competing household and economic demands that delay attendance for late vaccinations. Similar trends have been reported in other studies conducted in Kenya and across sub-Saharan Africa [10,13], which consistently show reduced timeliness for vaccines administered later in infancy and toddlerhood. Programmatic efforts should therefore focus on maintaining contact with caregivers throughout the vaccination schedule. Strengthening reminder systems, community-based follow-up, and flexible service delivery models, such as outreach clinics or extended service hours, could help mitigate delays and ensure completion of the immunization schedule on time.

Although children born to mothers with primary or higher education had slightly better timely vaccination across the antigens, the differences were not statistically significant. This suggests that in urban informal contexts, other factors such as mobility, access barriers, and health system constraints may play a greater role than maternal education alone. Male children showed a trend toward higher timely vaccination than female children for all the antigens, although the association was not statistically significant. This is consistent with earlier work in Nairobi’s informal settlements. For example, Janusz et al (2021) found equal representation of male and female children in vaccination card data and no significant sex‐based difference in vaccine timeliness [13].

This suggests that in this context, the gender of the child may not be a determinant of vaccination timing and that interventions might better focus on other barriers, such as the study site or access factors.

The association between caregivers’ religious affiliation and vaccination timeliness was most apparent for DTP-HIB-Hep-3 and MCV1, with comparatively smaller differences observed for the birth-dose BCG vaccine. While the reasons for these differences are not clear from our data, previous studies have shown that religious affiliation can sometimes influence health-seeking behaviors, including immunization practices [6,14]. In this context, faith communities may play an important role in shaping awareness of and engagement with vaccination programs. Collaborating with religious leaders and institutions could therefore support efforts to promote timely vaccination.

Timeliness varied significantly across survey rounds and sites. The first survey round recorded the highest proportion of untimely vaccinations, particularly for BCG and MCV1, while later rounds showed improvement. This pattern likely reflects the progressive recovery of immunization services following disruptions caused by the COVID-19 pandemic, which affected access and attendance at health facilities. Children from Viwandani had marginally higher timeliness of vaccination compared to those from Korogocho, possibly reflecting site-level differences in service availability, outreach efforts, or proximity to facilities. This is a unique finding as previous studies have not highlighted it. We attribute the finding to socio-demographic and contextual differences between these two urban informal settlements, as previously documented [15]. Viwandani has a predominantly youthful and working population, driven by its proximity to Nairobi’s industrial area. Residents of Viwandani have easy access to health information and services, as well as greater financial capacity, which enables them to access and use existing health services, including routine immunization. In contrast, Korogocho has a more stable population, but with larger household sizes and a higher dependency ratio compared to Viwandani. As a result, the cost of living in Korogocho is higher than in Viwandani, which may lead residents to prioritize basic needs such as food, water, and shelter over healthcare.

Overall vaccination timeliness in Nairobi’s informal settlements remains high, but gaps persist, particularly for vaccines administered later in infancy, such as MCV1.

These findings underscore the importance of sustained engagement with caregivers beyond the early immunization period and the need for adaptable delivery strategies that address competing socioeconomic and mobility challenges in urban informal settings.

### Strengths and limitations

The study has strengths and limitations. Examining the timeliness of BCG, DTP-HIB-Hep-3, and MCV1 administration provides insights into the performance of routine immunization services. Specifically, the findings shed light on access to immunization services after delivery, retention of children in immunization programs, the effectiveness of the link between the community and formal health systems, and the acceptability of routine immunization programs. These aspects have not been widely studied in informal settlements in Kenya or other sub-Saharan African settings. Another strength of the study concerns investigating the specific factors associated with the timeliness of administration of each vaccine, providing evidence for vaccine-specific interventions and cross-cutting interventions. However, there are some study limitations. The use of routine immunization cards as a primary data source may have excluded undocumented records, and this might have led to selection bias. However, our sensitivity analysis revealed no major systematic differences between included and excluded records for most measured characteristics, except for survey round and access to routine health services. We accounted for these differences in the multivariable models. Although some risk of selection bias remained, it was likely minimal. Moreover, the comparable socio-demographic characteristics between included and excluded records suggest that any residual bias is unlikely to have meaningfully influenced the observed associations.

Given the relatively small analytic sample and the high prevalence of timely vaccination, our ability to detect small but potentially meaningful differences may have been limited. Furthermore, despite adjusting for key socio-demographic variables, there are unmeasured confounders, and the findings demonstrate association but not causation since a cross-sectional design was used. Children who were excluded from the analytic sample were slightly older, more likely to have participated in earlier survey rounds, and had caregivers who more frequently reported uncertainty regarding access to routine health services. These characteristics may be associated with vaccination timeliness and could influence the observed associations.

Consequently, our findings may not be fully generalizable to all children in the surveillance population, and the magnitude of the reported associations may be over- or underestimated. Results should therefore be interpreted with caution, as vaccination timeliness may be overestimated among the analyzed sample relative to the broader study population. Timely vaccination appeared to vary by religious affiliation; however, the reference category (non-religious) included no children with untimely vaccination, limiting the reliability of comparisons. Despite these limitations, the study highlights the importance of addressing barriers to timely vaccine administration in urban informal settings. Future research should assess targeted interventions to improve vaccine timeliness in such areas, tailoring public health initiatives to the unique needs of informal settlements and improving child and maternal health outcomes.

## Conclusion and recommendation

Timeliness of vaccination among children below 5 years in urban informal settlements in Nairobi, Kenya, varied across the three vaccines, with the lowest timeliness observed for MCV1 and relatively high timeliness for BCG and DTP-HIB-Hep3. Differences across sites highlight the influence of local context, while religion emerged as a potential factor affecting timeliness. Maternal education and child gender showed minimal impact, suggesting structural and access barriers play a larger role. Strengthening outreach, reminder systems, and faith-based engagement can help improve timely vaccination in informal settlements.

## Supporting information

S1 File Inclusivity in global health research. (DOCX)

S1 Table Comparison of included and excluded records. (DOCX)

## References

[1] Nandi A, Shet A. Why vaccines matter: understanding the broader health, economic, and child development benefits of routine vaccination. Hum Vaccin Immunother. 2020;16(8):1900–4. doi: 10.1080/21645515.2019.1708669 31977283 PMC7482790

[2] World Health Organization. Vaccines and immunization. https://www.who.int/health-topics/vaccines-and-immunization#tab=tab_1. 2024.

[3] Bullen M, Heriot GS, Jamrozik E. Herd immunity, vaccination and moral obligation. 2023;2:636–41. 10.1136/jme-2022-108485 PMC10511978 37277175

[4] Zimba B, Mpinganjira S, Msosa T, Bickton FM. The urban-poor vaccination: Challenges and strategies in low-and-middle income countries. Hum Vaccin Immunother. 2024;20(1):2295977. doi: 10.1080/21645515.2023.2295977 38166597 PMC10766387

[5] Park JE, Kibe P, Yeboah G, Oyebode O, Harris B, Ajisola MM. Factors associated with accessing and utilisation of healthcare and provision of health services for residents of slums in low and middle- income countries: a scoping review of recent literature. 2022;1–18. 10.1136/bmjopen-2021-055415 PMC9125718 35613790

[6] KNBS. Demographic and Health Survey 2022. 2023.

[7] UNICEF. Immunization. https://data.unicef.org/topic/child-health/immunization/. 2024.

[8] Oduwole EO, Laurenzi CA, Mahomed H, Wiysonge CS. Enhancing routine childhood vaccination uptake in the Cape metropolitan district, South Africa: perspectives and recommendations from point-of-care vaccinators. 2022. 1–12. 10.3390/vaccines10030453 PMC8950960 35335085

[9] Kenya national immunization policy guidelines. Ministry of Health Kenya. 2023. http://health.go.ug/about-us/management

[10] Derqui N, Blake IM, Gray EJ, Cooper LV, Grassly NC, Pons-Salort M, et al. Timeliness of 24 childhood immunisations and evolution of vaccination delay: Analysis of data from 54 low- and middle-income countries. PLOS Glob Public Health. 2024;4(11):e0003749. doi: 10.1371/journal.pgph.0003749 39591390 PMC11593752

[11] Negash BT, Tediso Y, Yoseph A. Predictors of timeliness of vaccination among children of age 12-23 months in Boricha district, Sidama region Ethiopia, in 2019. BMC Pediatr. 2023;23(1):409. doi: 10.1186/s12887-023-04234-4 37598170 PMC10439539

[12] Masters NB, Wagner AL, Carlson BF, Boulton ML. Vaccination timeliness and co-administration among Kenyan children. Vaccine. 2018;36(11):1353–60. doi: 10.1016/j.vaccine.2018.02.001 29429814

[13] Janusz CB, Mutua MK, Wagner AL, Boulton ML. New Vaccine Introduction and Childhood Vaccination Timeliness in Two Urban, Informal Settlements in Nairobi, Kenya. Am J Trop Med Hyg. 2021;105(1):245–53. doi: 10.4269/ajtmh.21-0006 33999852 PMC8274788

[14] Melillo S, Strachan R, O’Brien CJ, Wonodi C, Bormet M, Fountain D. Effects of local faith-actor engagement in the uptake and coverage of immunization in low- and middle-income countries: A literature review. Christ J Glob Heal. 2022;9(1):2–32.

[15] Beguy D, Elung’ata P, Mberu B, Oduor C, Wamukoya M, Nganyi B, et al. Health & Demographic Surveillance System Profile: The Nairobi Urban Health and Demographic Surveillance System (NUHDSS). Int J Epidemiol. 2015;44(2):462–71. doi: 10.1093/ije/dyu251 25596586

