# Supplementary material for: Timeliness of vaccine administration among children in urban informal settlements in Nairobi, Kenya
Source: PLOS Glob Public Health. 2026 Feb 11;6(2):e0005462. doi: 10.1371/journal.pgph.0005462 (PMC12893561; doi:10.1371/journal.pgph.0005462)
Supplement: S1 Table — (DOCX) [file pgph.0005462.s002.docx]

**S1 Table:** **Comparison of included and excluded records.**

| **Variable** | **Level** | **Included records (n= 216)** | **Excluded records (n=196)** | **p-value** |
| --- | --- | --- | --- | --- |
| Study site | Korogocho | 82 (48.8) | 86 (51.2) | 0.220 |
|  | Viwandani | 134 (54.9) | 110 (45.1) |  |
| Sex | Female | 105 (52.5) | 95 (47.5) | 0.980 |
|  | Male | 111 (52.4) | 101 (47.6) |  |
| Religion | None | 3 (33.3) | 6 (66.7) | 0.440 |
|  | Muslims | 18 (58.1) | 13 (41.9) |  |
|  | Christians | 195 (52.4) | 177 (47.6) |  |
| Highest level of maternal education | No formal education | 194 (53.3) | 170 (46.7) | 0.330 |
|  | Primary and higher | 22 (45.8) | 26 (54.2) |  |
| Survey round | Round 1 | 38 (33.3) | 76 (66.7) | <0.001 |
|  | Round 2 | 52 (56.5) | 40 (43.5) |  |
|  | Round 3 | 62 (59.6) | 42 (40.4) |  |
|  | Round 4 | 64 (62.7) | 38 (37.3) |  |
| Unable to access routine health services during the COVID-19 pandemic in the last six months | Yes | 14 (43.8) | 18 (56.3) | 0.028 |
|  | No | 98 (60.5) | 64 (39.5) |  |
|  | Don’t know | 104 (47.7) | 114 (52.3) |  |
| Missed antenatal care at all during the last pregnancy | Yes | 1 (50.0) | 1 (50.0) | 1.000 |
|  | No | 215 (52.4) | 195 (47.6) |  |
| Missed delivery in a health facility | No | 216 (52.4) | 196 (47.6) |  |
